# Effects of Molecular Hydrogen in the Pathophysiology and Management of Cardiovascular and Metabolic Diseases

**DOI:** 10.31083/j.rcm2501033

**Published:** 2024-01-22

**Authors:** Ram B. Singh, Zuzana Sumbalova, Ghizal Fatima, Viliam Mojto, Jan Fedacko, Alex Tarnava, Oleg Pokotylo, Anna Gvozdjakova, Kristina Ferenczyova, Jana Vlkovicova, Branislav Kura, Barbora Kalocayova, Pavol Zenuch, Jan Slezak

**Affiliations:** ^1^Halberg Hospital and Research Institute, 244001 Moradabad, India; ^2^Pharmacobiochemical Laboratory of 3rd Department of Internal Medicine, Faculty of Medicine, Comenius University in Bratislava, 811 08 Bratislava, Slovakia; ^3^Era Medical College, Era University, 226003 Lucknow, India; ^4^3rd Department of Internal Medicine, Faculty of Medicine, Comenius University in Bratislava, 833 05 Bratislava, Slovakia; ^5^Department of Gerontology and Geriatric, PJ Safarik University, 040 86 Kosice, Slovakia; ^6^Drink HRW, Vancouver, BC 93030, Canada; ^7^Department of Food Biotechnology and Chemistry, Ternopil Ivan Puluj National Technical University, 46001 Ternopil, Ukraine; ^8^Center of Experimental Medicine, Institute for Heart Research, Slovak Academy of Sciences, 841 04 Bratislava, Slovakia

**Keywords:** antioxidant, free radical stress, endothelial dysfunction, dyslipidemia, diet, molecular hydrogen, inflammation

## Abstract

Diet and lifestyle choices, notably the Western-type diet, are implicated in oxidative stress and inflammation, factors that elevate the risk of cardiovascular diseases (CVDs) and type 2 diabetes mellitus (T2DM). In contrast, the Mediterranean of diet, rich in antioxidants, appears to have protective effects against these risks. This article highlights the dual role of diet in generating molecular hydrogen ($H_{2}$) in the gut, and $H_{2}$’s subsequent influence on the pathophysiology and prevention of CVD and T2DM. Dietary fiber, flavonoids, and probiotics contribute to the production of liters of $H_{2}$ in the gut, functioning as antioxidants to neutralize free radicals and dampen inflammation. In the last two decades, mounting evidence has demonstrated that both endogenously produced and exogenously administered $H_{2}$, whether via inhalation or $H_{2}$-rich water (HRW), have potent anti-inflammatory effects across a wide range of biochemical and pathophysiological processes. Recent studies indicate that $H_{2}$ can neutralize hydroxyl and nitrosyl radicals, acting as a cellular antioxidant, thereby reducing oxidative stress and inflammation—leading to a significant decline in CVDs and metabolic diseases. Clinical and experimental research support the therapeutic potential of $H_{2}$ interventions such as HRW in managing CVDs and metabolic diseases. However, larger studies are necessary to verify the role of $H_{2}$ therapy in the management of these chronic diseases.

## 1. Introduction

Obesity, type 2 diabetes mellitus (T2DM), and cardiovascular diseases (CVDs) are leading causes of global mortality [1, 2, 3]. These conditions share common risk factors including, an unhealthy diet, tobacco and alcohol use, and insufficient physical activity [1]. The impact of a western diet is notable, inducing oxidative stress compromising the body’s antioxidant capacity and instigating inflammation [4, 5]. This systemic inflammation can specifically affect the beta cells of the pancreas, hepatocyte low-density lipoprotein (LDL) receptors, endothelium, neurons, and osteocytes [6, 7]. These factors collectively raise the susceptibility to CVDs and T2DM, ultimately resulting in heightened mortality [6, 7].

The role of antioxidants in mitigating oxidative stress associated with these diseases remains an area of ongoing research. One hypothesis is that a healthy diet can balance oxidative stress levels, maintain cell and tissue homeostasis, and consequently reduce inflammation leading to a decreased risk of CVDs and metabolic disorders [8, 9]. Western diets (WD) exacerbate oxidative stress by elevating the levels of protein carbonylation and lipid peroxidation [4, 5] while decreasing the gut’s production of molecular hydrogen ($H_{2}$), a potential antioxidant [10, 11]. Conversely, Mediterranean diets (MD) which are rich in dietary fiber, flavonoids, and omega-3 fatty acids [12], may bolster antioxidant defenses by facilitating the production of protective molecules like $H_{2}$ [10, 11, 13].

While the protective mechanisms of $H_{2}$ remain unclear, evidence suggests that $H_{2}$ supplementation can reduce oxidative stress and inflammation, offering protection from CVDs and metabolic diseases [10, 11, 12, 13, 14, 15]. There are several methods for increasing $H_{2}$ including inhaling $H_{2}$ gas, drinking hydrogen-dissolved water ($H_{2}$-water), injecting hydrogen-dissolved saline ($H_{2}$-saline), taking hydrogen baths, and applying $H_{2}$-saline to the eyes. This communication aims to highlight the role of $H_{2}$, in the management of cardio-metabolic diseases (CMDs).

## 2. Free Radical Stress and Antioxidants in the Pathogenesis of Chronic Diseases

The combination of WD and environmental factors including pollution, tobacco smoke, pesticides and pollutants contribute to the generation of free radicals [4, 5, 10, 11, 14]. In the body inhaled oxygen ($O_{2}$) undergoes single electron reduction to form superoxide radicals ($O_{2}$^-^) [15]. These radicals can either propagate further oxidative reactions or transform into other reactive species such as hydrogen peroxide ($H_{2}$$O_{2}$) and hydroxyl radicals (•OH) [15]. Free radicals have an unpaired electron, and are consequently very reactive, requiring a single electron to form a stable electron shell [15]. These free oxygen radicals scavenge body tissues, leading to cellular and molecular damage [15]. This activity impacts cells, proteins, lipoproteins, and DNA, serving as a catalyst for various diseases [15].

The body naturally produces a range of free radicals, reactive oxygen species (ROS) and reactive nitrogen species from endogenous metabolic processes oxidants, exposure to environmental toxicities, and disease processes [15]. Maintaining a balance between free radicals and the body’s antioxidant defenses is critical for metabolic health, imbalances can elevate oxidative stress, causing tissue damage, and increasing the risk of conditions including CVDs and T2DM [8, 9, 10, 11, 14]. Interestingly, physiological levels of free radicals can have protective effects on cells, emphasizing the importance of endogenous antioxidants in neutralizing free radical-induced tissue damage [4, 5]. Notable endogenous sources of these toxins include xanthine oxidase, nicotinamide adenine dinucleotide phosphate (NADPH) oxidase, and electron leakage of electrons from the mitochondrial respiratory chain, which generate harmful superoxide radicals [16, 17].

## 3. Diet, Western Diet, Microbiome, and Molecular $H_{2}$

The WD, characterized by a high intake of saturated fats and sugar with a simultaneous low fiber intake, plays a significant role in the rise of chronic diseases and mortality rates [18]. Commonly consumed industrially manufactured ultra processed foods, including carbonated soft drinks, fast foods, industrially produced breads, or hot dogs have reduced nutritional values [19]. These dietary habits contribute to elevated cardiovascular risk factors (e.g., dyslipidemia, hypertension), and obesity or metabolic syndrome (MS) leading to increased mortality rate [18, 19, 20, 21]. This dietary pattern is increasingly becoming a health concern, driving a surge in metabolic diseases like diabetes and obesity, particularly in countries adopting a Western lifestyle [22].

Recent studies highlight the role of gut microbiota in the development and progression of inflammation, often called metaflammation which is linked to the development of non-communicable diseases involving immune system dysregulation [22]. Diets can affect the gut microbiota resulting in alterations to the host’s physiological responses. Consuming a WD can disrupt the gut’s microbial balance, leading to dysbiosis and further exacerbating gut inflammation [23].

In contrast, the MD is known for its anti-inflammatory properties, primarily due to its emphasis on a plant-based, prebiotic-rich foods, such as asparagus, garlic, onion, leeks, and omega-3 fatty acids [24]. These dietary components provide nutrients that interact with gut microbiota, and the immune system to maintain homeostasis [24]. Polyunsaturated fatty acids, fiber, and polyphenols may reduce the risk of chronic diseases by regulating oxidative stress [24]. The precursors of these diphenols are found in fiber-rich unrefined grain products, seeds, beans, peas, and berries [24]. While dietary antioxidants may provide protection from oxidative damage by neutralizing ROS, translating this into clinical practice has proven challenging. One issue is that antioxidants indiscriminately reduce all ROS levels, including those involved in beneficial physiological signaling [24].

## 4. Diet as Oxidant and Antioxidant Agent

**Fig. 1.**
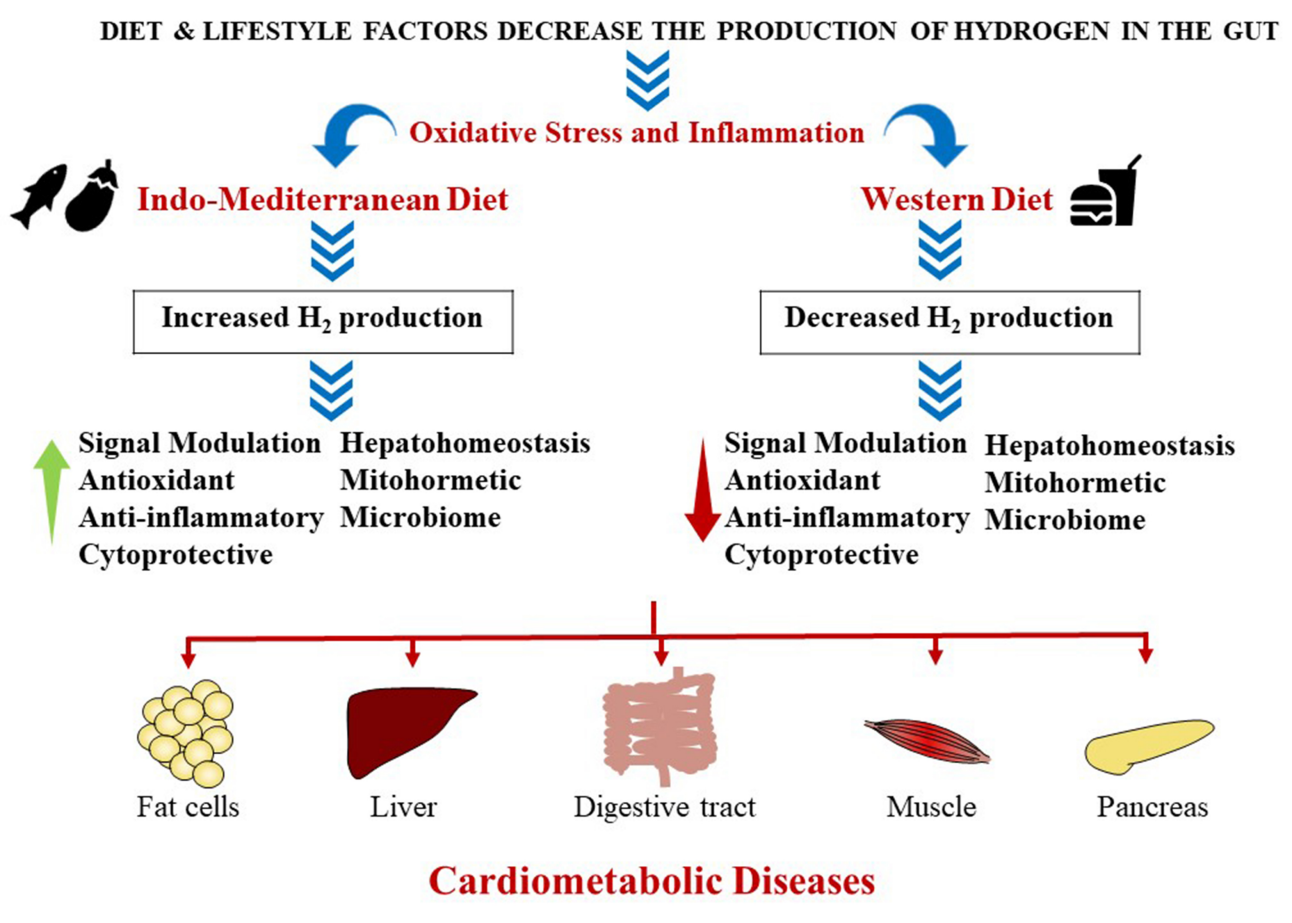
**The impact of diet on oxidative stress and inflammation: role of molecular hydrogen in of cardiovascular and metabolic disease development**. $H_{2}$, hydrogen.

The combination of a WD with low dietary antioxidant intake leads to an antioxidant deficiency, along with an increase in free radical stress-induced tissue damage throughout the body [4, 5, 10, 11, 14]. Noteworthy endogenous antioxidants such as catalase, superoxide dismutase, and ceruloplasmin are protective against damage to cholesterol receptors in the hepatocytes, beta cells of the pancreas, and endothelial damage by inhibiting free radical generation [8]. There is evidence that the WD is deficient in antioxidant nutrients such as flavonoids, fiber, and omega-3 fatty acids, leading gut microbes to produce fewer protective molecules including short chain fatty acids, glucagon like peptides, and $H_{2}$, which are potential anti-inflammatory agents [4, 5, 11, 14, 15]. Conversely, the MD is rich in antioxidants such as vitamins, minerals, flavonoids, omega-3 fatty acids, and fiber, can inhibit oxidative stress and inflammation, thereby reducing the risk of CVDs and T2DM [4, 5, 8, 9]. Antioxidant rich diets also promoted $H_{2}$ production in the gut, which may regulate circadian variations in blood pressure [8, 11, 14, 15]. In clinical settings, $H_{2}$ has been demonstrated to inhibit free radical stress in subjects with endothelial dysfunction, CVDs, and T2DM diabetes, that occur, due to oxidative stress [11, 15] (Fig. 1).

## 5. Production of Molecular Hydrogen in the Gut

**Fig. 2.**
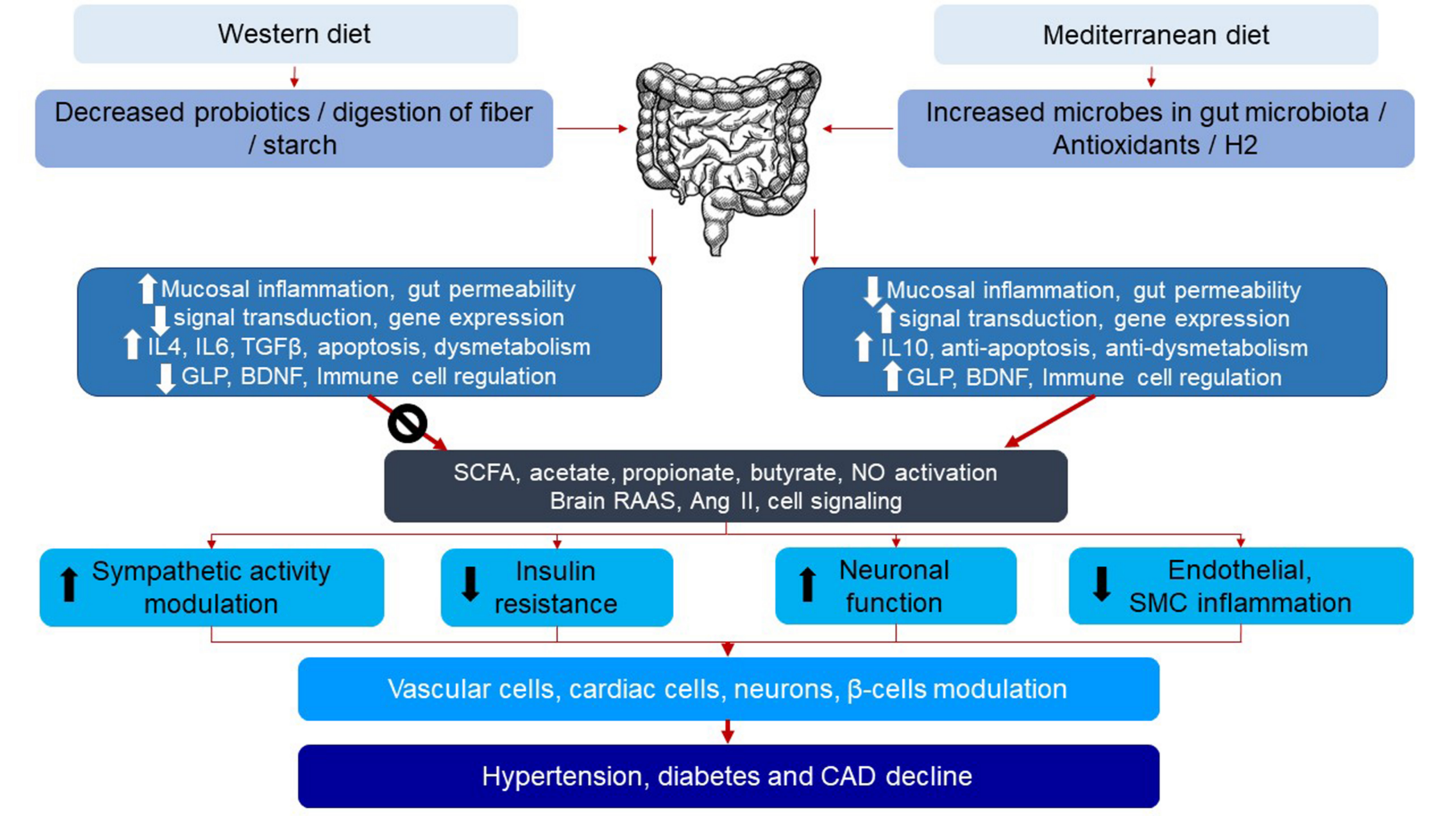
**Mechanism of production and inhibition of molecular hydrogen due to diets via microbiota in the gut, and its effects on anti-inflammatory molecules and cardio-metabolic diseases**. $H_{2}$, hydrogen; IL4, IL6, and IL10, interleukin 4, 6, and 10; TGFβ, transforming growth factor beta; GLP, glucagon-like peptide; BDNF, brain-derived neurotrophic factor; SCFA, short-chain fatty acids; NO, nitric oxide; RAAS, renin-angiotensin-aldosterone system; Ang II, angiotensin II; CAD, coronary artery disease; SMC, smooth muscle cell.

The gut microbiota plays a crucial role in mitigating the risk of T2DM and CVDs [25]. Many complex carbohydrates and plant polysaccharides escape digestion in the gut due to the absence of enzymes [25]. However appropriate microbes can metabolize these polysaccharides into beneficial short-chain fatty acids (SCFAs) with potential anti-inflammatory effects such as propionate, butyrate, and acetate, along with gases like methane and $H_{2}$ [25, 26]. $H_{2}$ is generated through fermentation of carbohydrates – such as lactose, lactulose, and fructose by intestinal bacteria [25, 26]. The primary bacteria involved in $H_{2}$ production are groups such as *Bacteroides fragilis*, *Clostridium perfringens*, and *Pseudomonas*, all of which are normally present in the large intestine and possess hydrogenases [25, 26]. This fermentation producing SCFA typically occurs in the colon [25, 26]. Their concentration tends to be higher in the proximal colon and lower in the distal colon, despite the latter having a greater microbial density and elevated gas levels [25, 26]. Besides their local effects, SCFAs like acetate can influence neural function, offering a potential pathway for gut-brain interactions [25, 26] (Fig. 2).

$H_{2}$ production in the human gastrointestinal (GI) tract is primarily dependent on the fermentation of ingestible fibrous substrates by a rich intestinal flora [27], predominantly located in the colon [24]. The amount of endogenous $H_{2}$ produced through this mechanism generally surpasses that obtained from consuming $H_{2}$-rich water (HRW). Excess $H_{2}$ can be removed through multiple microbial pathways [11]. In addition to methanogenesis, another mechanism involves sulphate-reducing bacteria using excess $H_{2}$ to convert sulphate to sulfite. The “keystone pathogen” hypothesis offers an explanation for the role of specific microbes in disease states, that certain low-abundance microbial pathogens can disrupt a normally benign microbiota, converting it into a disease associated, or dysbiotic state [28]. These pivotal microbes, termed “keystone pathogens”, play a role in creating an environment conducive to disease, particularly by fostering inflammation [29]. Recent studies substantiate the idea that these pathogens instigate disease by altering the gut microbiota [29].

When combined with the proper diet, gut microbiota can generate between 3-9 liters of $H_{2}$ in the colon [30]. $H_{2}$ is formed as an end product of polymeric carbohydrate fermentation caried out by members of the *Firmicutes* and *Bacteroidetes* microbial taxa [30]. There are two primary pathways for $H_{2}$ disposal, methanogenesis, and homoacetogenesis, with the latter being more predominant. $H_{2}$ is produced by many members of the gut microbiota and may be subsequently utilized by cross-feeding microbes for growth and in the production of larger molecules [31]. $H_{2}$ can serve as a substrate for hydrogenotrophic microbes, which fall into three categories: sulfate-reducing bacteria, methanogenic archaea, and acetogenic bacteria, which can convert $H_{2}$ into hydrogen sulfide, methane, and acetate, respectively [30, 31, 32]. It is becoming increasingly clear that $H_{2}$ plays a crucial role in GI microbial metabolism, impacting human nutrition, health, and wellbeing, with a growing body of evidence suggesting a strong correlation between the volume of $H_{2}$ production by intestinal bacteria and various diseases [13].

A pilot study reported that consumption of $H_{2}$-producing milk four hours prior to exercise significantly decreased creatine kinase and 8-hydroxy-2-deoxyguanosine levels while improving muscle recovery following exercise [33]. Previous research indicated that acetate facilitates a microbiome–brain–β-cell axis that exacerbates MS [26], while increased production of metabolites including short chain fatty acids, brain-derived neurotrophic factor (BDNF), and $H_{2}$ enhance metabolism via gut-brain interaction neural circuits [25]. A healthy gut microbiota is promoted when the fiber rich MD includes probiotics, increasing $H_{2}$ production to levels measurable in liters [27, 28, 29]. The medical community is increasingly exploring natural, non-toxic compounds like $H_{2}$ for their potential antioxidative roles in preventing cardiovascular diseases and other chronic conditions [10, 11, 14, 15]. The evolving understanding of the biological importance of intestinal $H_{2}$ has shifted the perception of its significance. No longer just a byproduct, $H_{2}$ is now viewed as a critical factor in global organ function and homeostasis [11, 15, 16, 17].

**Fig. 3.**
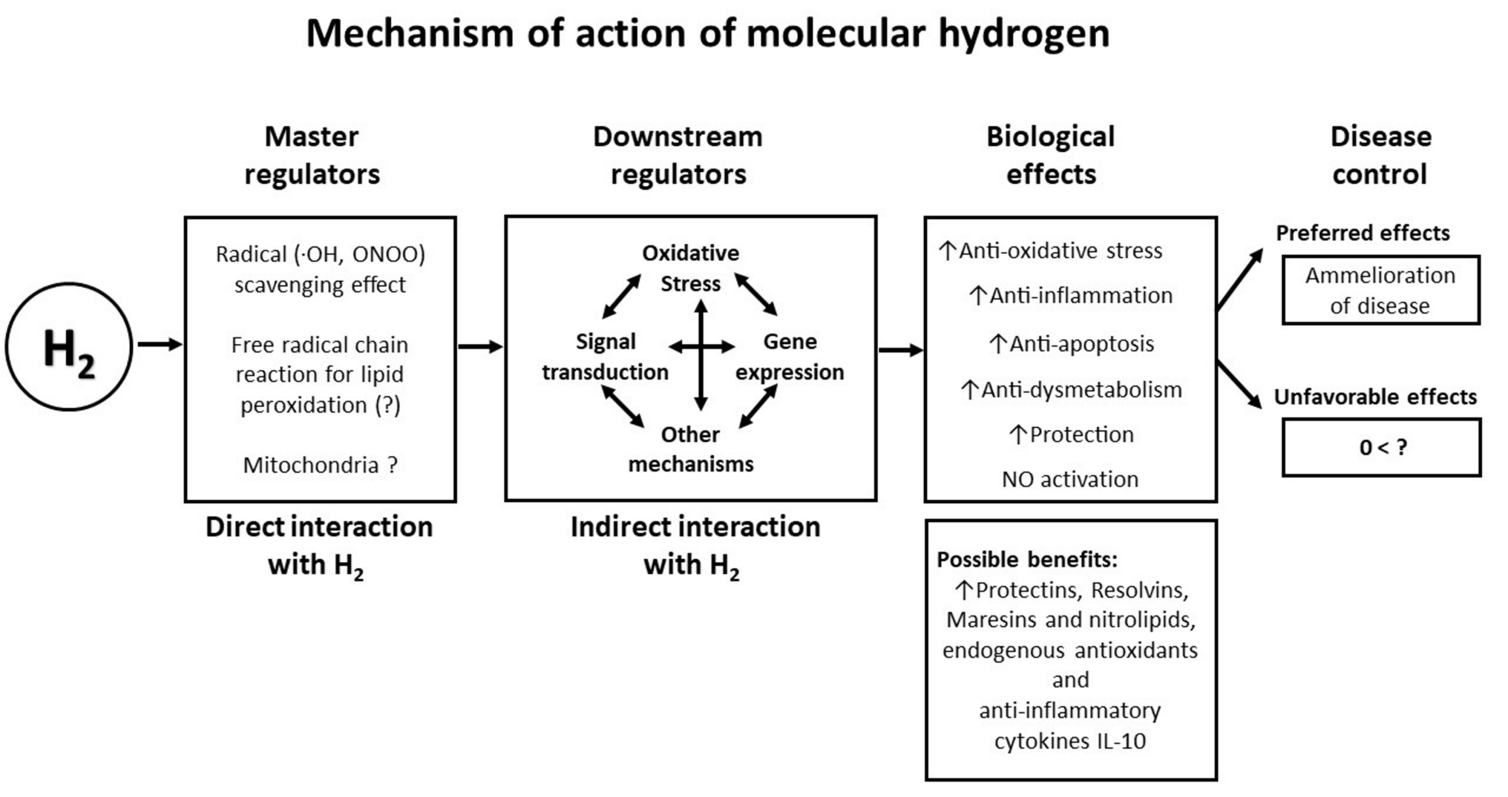
**Mechanism of action of molecular hydrogen in the pathogenesis and control of cardiovascular and metabolic diseases (Modified from reference [11], Ichihara *et al*., Med Gas Res 2015)**. •OH, hydroxyl radical; ${\mathrm{ONOO}}^{-}$, peroxinitrite; $H_{2}$, hydrogen; NO, nitric oxide; IL-10, interleukin 10.

Over the past two decades $H_{2}$ has emerged as a versatile antioxidant with applications across a spectrum of physiological and pathophysiological conditions. Whether endogenously produced through healthy foods or exogenous administration via inhalation or HRW, $H_{2}$ has shown promise as a potential antioxidant in a wide range of physiological and pathophysiological processes [16, 17]. $H_{2}$ can inhibit hydroxyl and nitrosyl radicals in the cells and tissues, causing a marked decline in oxidative stress, leading to a decline in the inflammation that is marker in the pathogenesis of diabetes and CVDs [17]. Interestingly, Slezák *et al*. [10] and other researchers [11, 14, 15] have demonstrated that $H_{2}$ can rapidly diffuse into tissues and cells without disrupting metabolic redox reactions or signaling reactive species (Fig. 3, Ref. [11]).

In addition to regulating gene expression, $H_{2}$ engages in epigenetic modulation, offering alternative pathways for mitigating oxidative stress-induced genetic damage, thereby enhancing its anti-inflammatory and anti-apoptotic capabilities [16, 17]. $H_{2}$ also alleviates blood-brain barrier impairment and improves cognitive dysfunction [34]. Hydrogen therapy has been found to ameliorate cardiac remodeling [35], dyslipidemia and MS [36] oxygen saturation in chronic lung disease [37], and in nonsteroidal anti-inflammatory drug (NSAID)-induced enteropathy [38].

## 6. Oxidative Stress, Inflammation, Immunomodulation, and Effects of $H_{2}$

Oxidative stress arises from an the imbalance between the production of reactive oxygen and nitrogen species and the body’s ability to eliminate reactive intermediates. Many antioxidants acting through different mechanisms have been successfully used as a form of therapy, preventing cell damage [10, 39, 40]. While oxidative stress is a natural part of aging, over 2000 scientific papers implicate chronic oxidative stress to the development of a whole range of chronic pathological conditions [39]. Critical macromolecules including DNA, proteins, and membranes can be damaged by highly reactive hydroxyl and nitrosyl radicals during periods of oxidative stress [39, 41, 42].

•OH radicals are highly reactive and can interact with virtually any biological molecule in the vicinity [41]. The scavenging of free radicals can serve both preventive and therapeutic roles [39]. $H_{2}$, due to its selective reactivity, stands out as a unique scavenger, reacting only with •OH and peroxynitrite (${\mathrm{ONOO}}^{-}$) [39]. Other ROS like superoxide ($O_{2}$•^-^), $H_{2}$$O_{2}$ and nitric oxide (•NO)—which also serve as signaling molecules—remain unaffected [39]. Furthermore, $H_{2}$ indirectly regulates hormones and cytokines through various signal transduction pathways [39]. During the inflammatory response immune cells break the homeostatic balance; $H_{2}$ inhibits pro-inflammatory signaling and activates anti-inflammatory signaling [43].

An essential attribute of $H_{2}$ is its permeability, enabling rapid penetration of the cell membrane and dispersion throughout the cytoplasm, nucleus, and other organelles to confer protective effects [44]. In contrast to most antioxidant compounds, $H_{2}$ can pass through the blood–brain barrier, and thus far, there have been no reports of cytotoxicity [44]. $H_{2}$ also has no direct effect on body temperature, blood pressure, pH, or ${\mathrm{pO}}_{2}$ [44]. $H_{2}$ exerts anti-inflammatory and antioxidative effects by directly interacting with the mitochondrial electron transport chain and neutralizing oxidative stress [45]. Overall, this alleviates mitochondrial damage, balances intracellular environmental homeostasis, and protects the transcription of key regulatory proteins of inflammation [45].

## 7. Molecular Hydrogen Therapy for Cardiovascular Diseases

Damage to cardiomyocytes, vasculature—including endothelium and smooth muscle cells—are all CVD risk factors that result in cardiovascular dysfunction [35]. Increases in fibrosis and apoptosis are closely related heart failure [35]. Therefore, novel therapeutic approaches for the treatment of cardiac remodeling and fibrosis of myocardium are needed to improve the survival rates of patients with cardiac ischemia. In a rat model of myocardial infarction, $H_{2}$ treatment (inhalation of 2% $H_{2}$ for 28 days daily for 3 hours) significantly improved cardiac function while decreasing the area of fibrosis [35]. Complementary *in vitro* experiments also revealed that $H_{2}$ therapy mitigated hypoxia-induced damage to cardiac cells and inhibited the angiotensin II-induced migration and activation of cardiac fibroblasts [35].

ROS play a significant role in vascular disease development while also modulating blood vessel vasomotor function [14]. These free radicals neutralize •NO, converting it into the more harmful peroxynitrite radical [43]. NADPH oxidase (NOX) family proteins, the oxidases that produce $H_{2}$$O_{2}$ and superoxide, are the main source of vascular free radicals [17, 43, 46, 47]. Variations in blood pressure and flow can impact endothelial function, which is crucial for maintaining vasomotor tone, as the arterial endothelium actively modulate shear stress [14]. Accumulated oxidative stress and inflammation can lead to endothelial dysfunction, predisposing individuals to atherosclerosis and CVDs [14]. Endothelium-derived relaxing factors (EDRF), such as •NO, endothelium-derived hyperpolarization factor (EDHF) and prostacyclin are known to play a crucial role in the development of diet induced vascular dysfunction [4, 5]. The shear stress activates the NOX proteins—specifically NOX 1, NOX2 and NOX3 —which are key factors in vascular function [46, 47]. Superoxide radicals, primarily generated by NOX1 and NOX2 through single electron transfer to $H_{2}$, rapidly neutralize excess •NO within cells, leading to the production of peroxynitrite [47]. This compound adversely affects vasodilation mediated by nitric oxide [47].

In the presence of peroxynitrite, an indication of oxidative dysfunction, there may be a suppression of endothelial nitric oxide synthase (eNOS) enzyme activity, leading to reduced NO production [47]. The eNOS oxidation inducing cofactor, tetrahydrobiopterin (BH4), may be converted to the inactive form 7,8-dihydropterin (BH2). This conversion leads to the uncoupling of eNOS, a mechanism that generates superoxide radicals [47].

The redox imbalance between •NO and superoxide radical production in endothelial cells may lead to endothelial dysfunction [14]. Another ROS, $H_{2}$$O_{2}$ have both detrimental and beneficial effects on vascular function. Although the role of the hydroxyl radical—a byproduct of hydrogen peroxide decay—is not fully understood, it is known to impair endothelial function. This impairment can be counteracted by $H_{2}$ [14]. A clinical study demonstrated that $H_{2}$ therapy significantly improves flow-mediated dilatation (FMD) in healthy volunteers suggesting protective effects on vascular function [14]. In the group receiving high levels of $H_{2}$, FMD increased from 6.80% ± 1.96% to 7.64% ± 1.68% (mean ± SD) compared to a decrease from 8.07% ± 2.41% to 6.87% ± 2.94% in the placebo group [14]. These findings indicate that $H_{2}$ may protect vascular tissues from damage induced by shear stress and hydroxyl radicals, while preserving the beneficial effects of nitric oxide on vasomotor function. Given that oxidative stress can exacerbate systemic inflammation and thereby impair the function of cardiomyocytes, beta cells, and neurons, as well as endothelium, the potential protective roles of $H_{2}$ warrant further investigation [48, 49, 50, 51, 52].

**Fig. 4.**
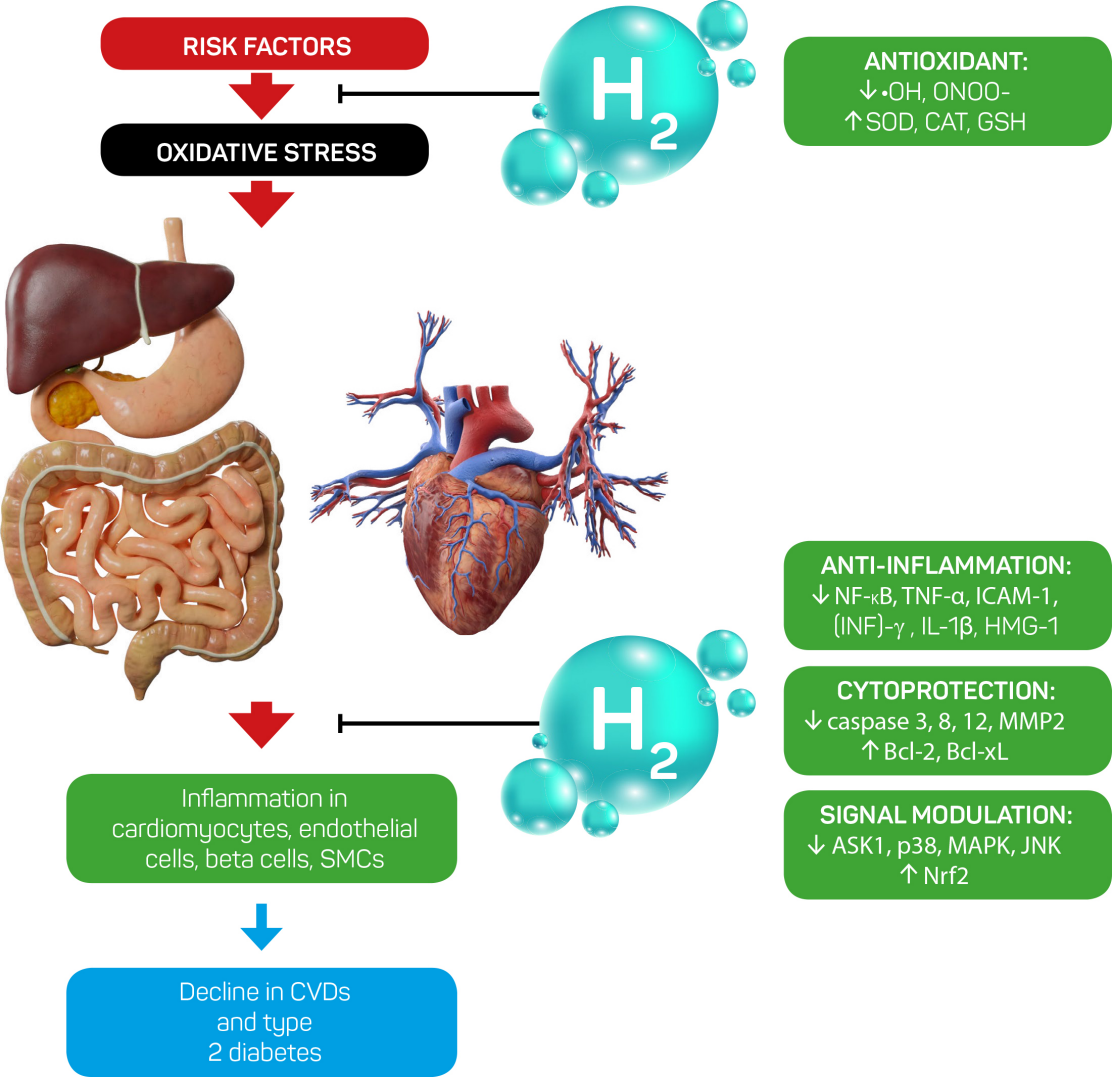
**Mechanisms of the effects of hydrogen therapy on cardio-metabolic diseases (Modified from LeBaron *et al*., 2019, reference [42])**. SMCs, smooth muscle cells; CVDs, cardiovascular diseases; NF-κB, nuclear factor kappa B; TNF-α, tumor necrosis factor alpha; ICAM-1, inter cellular adhesion molecule-1; (INF)-γ, interferon gamma; IL-1β, interleukin 1 beta; HMG-1, high mobility group box 1 protein; MMP2, matrix metalloproteinase 2; Bcl-2, B-cell lymphoma 2; Bcl-xL, B-cell lymphoma-extra large; ASK1, apoptosis signal-regulating kinase 1; MAPK, mitogen-activated protein kinase; JNK, c-Jun N-terminal kinases; Nrf2, nuclear factor erythroid 2-related factor 2; •OH, hydroxyl radical; ${\mathrm{ONOO}}^{-}$, peroxinitrite; SOD, superoxide dismutase; CAT, catalase; GSH, glutathione; $H_{2}$, hydrogen.

ROS are generated as essential co-factors during oxidative phosphorylation via electron transfer, a process that occurs in aerobic metabolism [11, 14, 15]. Rheumatoid arthritis (RA) is known to elevate the risk of coronary artery disease (CAD) and atherosclerosis, which in turn increases mortality from CVD [53]. This link can be attributed to overlapping inflammatory pathways in both RA and CAD [53]. Specifically, free radicals and pro-inflammatory cytokines appear to be key drivers connecting these diseases [54]. These inflammatory mechanisms impact both the vascular endothelium and joint tissues in arthritis. Endothelial and smooth muscle cells produce superoxide radicals through NADPH oxidases, including NOX1, NOX2, NOX4, and NOX5, which are crucial to endothelial function and progression of atherosclerosis [53, 54, 55]. The oxidation of low-density lipoprotein cholesterol (LDL-C), observed as an intersection between these mechanisms, predisposing plaque development in atherosclerosis, consequently leading to high CVD risk [56, 57]. The development of CAD or stroke in patients with arthritis may lead individuals to be predisposed due to changes in endothelial phenotypic response to a high quantity of harmful stimuli. Oxidative stress upregulates the expression of adhesion molecules such as vascular cell adhesion molecule-1 (VCAM-1), intercellular adhesion molecules 1 (ICAM-1), and E-selectin. The pro-inflammatory cytokines tumor necrosis factor alpha (TNF-α), interleukin 1 (IL-1), interferon-γ, are also activated in the pathogenesis of atherosclerosis [53, 54, 55, 56, 57]. Interestingly, vascular dysfunction may also occur due to up-regulation of TNF-α expression alone, leading to atherosclerosis [58]. In patients with rheumatoid arthritis, anti-TNF-α therapies could reduce the progression of atherosclerosis, indicating that the pathogenesis of atherosclerosis involves shared TNFα/ROS inflammatory pathways at the crossing between Loop 1 and 2 [58]. Further studies by Slezak and his group [59, 60, 61, 62] have illustrated the role of $H_{2}$ in hypoxic post-conditioning, radiation-induced heart injury, or acute cardiac injury (Fig. 4, Ref. [42]).

## 8. Effects of Hydrogen in Stroke

The medicinal value of $H_{2}$ has been shown by inhalation of 2% $H_{2}$ which can significantly decrease the damage caused during cerebral ischemia/reperfusion, which in turn are caused by oxidative stress via selective elimination of •OH and ONOOˉ [63, 64, 65, 66, 67, 68, 69, 70]. Numerous experimental and clinical studies involving $H_{2}$ indicate that therapy produces anti-oxygenation, anti-inflammation, and anti-apoptosis effects. Since brain tissue is highly susceptible to cell damage, produced by free radicals and other markers, $H_{2}$ therapy benefits may be easier to demonstrate in patients predisposed to stroke [63, 64, 65, 66, 67, 68, 69, 70]. A single comprehensive review accounting for the blood-brain barrier, penetrability, possible side effects, and the molecular properties of $H_{2}$, should contribute to advancing both clinical and experimental research and therapies. In clinical studies, upon ischemic stroke onset, 8.5–30% of patients suffer a hemorrhagic stroke, and the rest experience an ischemic stroke [64]. In animal studies, small doses of $H_{2}$ have been shown to significantly reduce mortality in cases of brain wide ischemic strokes [59]. When $H_{2}$ was administered to groups with high sugar levels and transient middle cerebral artery occlusion (tMCAO), it effectively lowered the risk of brain hemorrhage [59]. Sustained inhalation of 2.9% $H_{2}$ for 2 hours led to a significant reduction in oxidation and nitration byproducts, as well as in matrix metalloproteinase-9 (MMP-9), suggesting that the blood-brain barrier was better preserved [59]. Chen *et al*. [66] proposed that this effect contributed to the lower occurrence of hemorrhage accompanying cerebral infarction. In a separate study, mice were subjected to global cerebral ischemia/reperfusion (I/R) through a 45-minute occlusion of both common carotid arteries (BCCAO) [64]. Inhalation of 1.3% $H_{2}$-rich air improved the 7-day survival rate, significantly mitigating neuronal damage, autophagy in the hippocampal CA1 region, and brain edema. Additionally, the administration of $H_{2}$ led to lower levels of oxidative stress markers ${\mathrm{8-hydroxy-2}}^{\prime }$-deoxyguanosine (8-OHdG) and malondialdehyde in brain tissues [64].

A hemorrhagic stroke is defined as a cerebral hemorrhage followed by compression and necrosis of brain tissue [67]. Hemorrhagic strokes are typically more dangerous than ischemic strokes because they involve microglia and inflammatory cells, which are activated upon hemorrhage, producing free radicals [68]. In a mouse model of intracerebral hemorrhage, inhalation of 2% $H_{2}$ for one hour significantly reduced the degree of cerebral edema and significantly improved neural function [69]. Interestingly, these improvements were limited to 72 hours, suggesting that $H_{2}$ inhalation provides protection only in the acute phase of cerebral hemorrhage [69]. The delay in the peak of neutrophil infiltration and microglial activation, occurring after 72 hours, might explain why the anti-oxygenation effects of $H_{2}$ were not sufficiently persistent during that period [69]. Additionally, $H_{2}$’s protective effect on the blood-brain barrier and its ability to reduce cerebral edema may be attributed to its moderating influence on mastocyte activity, which is crucial in the initial inflammatory responses following a stroke [69, 70]. In a study involving rats with acute hyperglycemia, treatment with $H_{2}$-rich saline was associated with increased hemorrhagic transformation in a focal ischemia [66]. Meanwhile, a rabbit model of subarachnoid hemorrhage on brain stem infarction showed the combination of $H_{2}$ and edaravone treatment led to a more significant reduction in recovery time compared to using edaravone alone [70].

## 9. Effects of Molecular Hydrogen on Blood Lipoproteins

Increased concentrations of blood lipids and pro-inflammatory cytokines are risk factors of CVDs. Clinical and experimental studies indicate that $H_{2}$ administration has beneficial lipid-lowering effects. In a case study of 20 patients with MS, HRW (0.9–1.0 L/day) was administered to determine its effects on biological activities of serum lipoproteins [28, 71]. Following 10 weeks of treatment, HRW produced a decline in total-cholesterol (TC) and LDL-C concentrations [28, 71]. This was accompanied by a significant decline in apolipoprotein (apo)E, apoB100, and an improvement in function of high-density lipoprotein (HDL) [28, 71]. The intake of HRW was associated with rise in superoxide dismutase (SOD) and a reduction in thiobarbituric acid-reactive substances (TBARS) in the LDL and serum, important markers of MS [28, 71]. In a clinical trial, 68 patients with high cholesterol were randomized to either HRW (0.9 L/day, n = 34) or placebo (n = 34) for a total period of 10 weeks [29, 72]. In the group treated with HRW, the isolated HDL cholesterol demonstrated enhanced efficacy in promoting adenosine triphosphate (ATP)-dependent cholesterol efflux, specifically related to the cassette transporter A [29, 72]. Concurrently, there was an increase in plasma levels of pre-β-HDL, while HDL-cholesterol concentrations remained unchanged [29, 72]. Moreover, HRW treatment was associated with improvement in other HDL functions related to LDL oxidation, specifically inhibition of pro-inflammatory oxidized-LDL and the protection of endothelial cells from apoptosis. In addition, therapy with HRW was associated with the improved down-regulation of total cholesterol (47.06% vs. 17.65%) and LDL-C (47.06% vs. 23.53%). There was a significant decline in apoB100 with rise in apoM in the $H_{2}$ group. Treatment with $H_{2}$ was associated with a marked decline in the concentrations of multiple pro-inflammatory markers as well as indicators of oxidative stress in the plasma and HDL particles. The present results emphasize the potential efficacy of $H_{2}$ therapy in the reduction of cholesterol as well as atherosclerosis.

## 10. Effects of Molecular Hydrogen in Diabetes Mellitus and Metabolic Syndrome

MS is characterized by the presence of at least three of the following risk factors including obesity, diabetes, hypertension, hyperlipidemia, and low HDL [71]. Free radicals, with or without inflammation, are thought to play key roles in the development of MS and T2DM [71, 72, 73]. Therapy with HRW has shown promise in improving glucose and lipid metabolism in individuals with T2DM or glucose intolerance, conditions which are both linked to oxidative stress [73, 74]. The effectiveness of HRW (1.5–2 L/day) was examined in an open label, 8-week study in 20 subjects with potential MS [73]. HRW was generated by inserting a metallic magnesium stick into drinking water, leading to an $H_{2}$ concentration between 0.55–0.65 mM) produced from the following chemical reaction [73]:

*Mg + ${\mathrm{2H}}_{2}$O → Mg ${\mathrm{(OH)}}_{2}$ + $H_{2}$*

The consumption of HRW for 8 weeks resulted in a 39% increase (*p* < 0.05) in the antioxidant enzyme SOD and a 43% decrease (*p* < 0.05) in TBARS in urine [73]. Furthermore, subjects showed an 8% increase in HDL-cholesterol and a 13% decrease in total cholesterol/HDL-cholesterol from baseline to week 4 [73]. There was no change in fasting glucose concentrations during the 8-week study [73]. Drinking HRW may represent a potentially novel therapeutic and preventive strategy for MS.

Singh and colleagues [36] conducted a randomized, placebo-controlled trial where patients with MS with were treated with HRW, showing favorable effects on multiple parameters following 24 weeks when compared to placebo group (*p* < 0.05, *p* = 0.309). The results were accompanied by significant declines in body mass index (BMI) and waist-to-hip ratio (WHR, *p* < 0.05) [36]. In addition, treatment with HRW caused a significant decline in blood lipids as shown in Table 1 (Ref. [36]).

**Table 1.** **Effects of hydrogen rich water on blood lipoproteins in patients with metabolic syndrome [36]**.

Data	Hydrogen rich water (n = 30)		Placebo (n = 30)	
Data, mg/dL	Baseline	After 24 weeks	Baseline	After 24 weeks
Cholesterol	187.7 ± 32.4	169.2 ± 26.1***	184.3 ± 37.4	184.4 ± 38.6
LDL cholesterol	109.0 ± 34.4	102.5 ± 28.0	105.5 ± 42.0	106.0 ± 43.3
HDL cholesterol	41.7 ± 4.2	40.4 ± 1.8	41.8 ± 2.3	42.3 ± 2.4
VLDL cholesterol	37.3 ± 17.9	28.0 ± 11.3**	36.8 ± 20.6	37.3 ± 20.5
Triglycerides	189.8 ± 93.3	142.4 ± 65.0**	184.4 ± 102.8	185.6 ± 101.3
C-reactive proteins	0.5 ± 0.2	0.5 ± 0.1*	0.6 ± 0.5	0.6 ± 0.5

*** = *p* value < 0.0001, ** = *p* value < 0.01, * = *p* value < 0.05, by comparison of baseline and after following up using analysis of variance (Modified from reference [36]). LDL, low density lipoprotein; HDL, high density lipoprotein; VLDL, very-low-density lipoprotein.

Treatment with HRW also reduced fasting blood glucose after 24 weeks, along with a significant decline in glycated haemoglobin (HbA1C) (12%, *p* < 0.05) compared to baseline levels and placebo group [36]. Treatment with HRW also reduced the markers of inflammation: TNF-α, and IL-6 (*p* < 0.05) [36]. While oxidation markers showed a significant decline, there were increases in vitamins C and E in the $H_{2}$ group [36]. Serum levels of angiotensin converting enzyme were significantly decreased whereas serum nitrite level showed significant increases (Table 2, Ref. [36]), which may lead to declines in blood pressure.

**Table 2.** **Effect of hydrogen rich water on glycaemia, oxidative stress, and cytokines in patients with metabolic syndrome [36]**.

	Hydrogen rich water (n = 30)		Placebo (n = 30)	
Data, mg/dL	Baseline	After 24 weeks	Baseline	After 24 weeks
Fasting blood glucose	121.5 ± 61.0	103.1 ± 33.0*	123.9 ± 43.4	126.4 ± 42.3
HbA1c, %	5.8 ± 0.9	5.1 ± 0.2***	6.2 ± 1.2	6.1 ± 1.2
TNF-α	4.8 ± 1.2	3.9 ± 0.6***	4.8 ± 1.3	4.8 ± 1.3
IL-6	1.9 ± 0.7	1.6 ± 0.2**	1.6 ± 0.6	1.7 ± 0.6
TBARS	2.5 ± 0.3	1.6 ± 0.3*	2.5 ± 0.3	2.5 ± 0.3
Malondialdehyde	3.4 ± 0.2	2.7 ± 0.2***	3.4 ± 0.2	3.5 ± 0.2
Diene conjugates	27.8 ± 1.0	26.7 ± 0.5***	28.3 ± 0.8	28.3 ± 0.8
Vitamin E	23.0 ± 2.3	26.8 ± 1.9***	23.0 ± 1.5	23.1 ± 1.1
Vitamin C	20.7 ± 2.5	24.2 ± 1.8***	20.7 ± 2.5	20.8 ± 2.4
Nitrite	0.63 ± 0.06	0.68 ± 0.06***	0.66 ± 0.04	0.65 ± 0.03
Angiotensin converting enzyme	85.2 ± 7.8	80.7 ± 5.8***	84.5 ± 8.8	83.8 ± 8.7

*** = *p* value < 0.0001, ** = *p* value < 0.01, * = *p* value < 0.05, by comparison of baseline and after follow up using analysis of variance (Modified from reference [36]). HbA1c, glycated hemoglobin; TNF-α, tumor necrosis factor alpha; IL-6, interleukin 6; TBARS, thiobarbituric acid reactive substances.

In a randomized, controlled, cross-over trial involving 30 patients with T2DM and 6 patients with impaired glucose tolerance, patients took either 900 mL/d of HRW or 900 mL of placebo water for 8 weeks, with a 12-week period of washout [74]. Intake of HRW led to significant declines in modified LDL-C (i.e., modifications that increase the net negative charge of LDL), small dense LDL, and urinary 8-isoprostanes by 15.5% (*p* < 0.01), 5.7% (*p* < 0.05), and 6.6% (*p* < 0.05), respectively [74]. Additionally, there was a trend towards lower serum concentrations of oxidized LDL and free fatty acids, as well as increased plasma concentrations of adiponectin and extracellular SOD [74]. These results suggest that HRW could be a useful adjunct in preventing T2DM and insulin resistance, potentially by activating ATP-binding cassette transporter A1-dependent efflux and enhancing the anti-atherosclerotic functions of HDL, and have beneficial lipid-lowering effects [74]. These findings suggest that HRW could be a useful adjunct in preventing T2DM and insulin resistance, potentially by activating ATP-binding cassette transporter A1-dependent cholesterol efflux and enhancing the anti-atherosclerotic functions of HDL [74]. Since MS has become a worldwide problem, $H_{2}$ therapy may be a new approach for mitigating CMDs [73, 74, 75, 76, 77]. A recent review has also reemphasized that Indo-Mediterranean diets can produce greater $H_{2}$, and may be a better option for preventing hypertension [78].

$H_{2}$ therapy may have a beneficial impact on mitochondrial function, as shown in a rat study conducted by Gvozdjáková *et al*. [79]. The study showed that administering $H_{2}$ to rats led to enhanced state 3 respiration in cardiac mitochondria, linked to both Complex I (CI) and Complex II (CII) substrates [79]. It was proposed that $H_{2}$ may facilitate the conversion of quinone intermediates in the Q-cycle to the fully reduced ubiquinol [80]. This conversion could boost the antioxidant capacity of the quinone pool, thereby reducing the generation of mitochondrial ROS [80].

## 11. Conclusions

The past two decades have seen increased interest in the potential health benefits of $H_{2}$, particularly in cardiovascular and metabolic diseases. The primary mechanism behind $H_{2}$’s therapeutic effects appears to be its selective and efficient scavenging and neutralization of ROS such as •OH and •${\mathrm{ONOO}}^{-}$. Beyond its antioxidant role, $H_{2}$ also exhibits anti-inflammatory and anti-apoptotic properties. Our review indicates that $H_{2}$ administration shows promise in mitigating CVDs, atherosclerosis, stroke, and hyperlipidemia, with potential applicability in coronary artery disease and diabetes. Notably, $H_{2}$ can be endogenously produced in the human gut by specific bacteria, a process that can be optimized through dietary choices. For example, a Mediterranean-style diet, rich in fiber and bioactive compounds, may enhance gut-based $H_{2}$ production. Our review indicates that $H_{2}$ administration shows promise in mitigating CVDs, atherosclerosis, stroke, and hyperlipidemia, with potential applicability in coronary artery disease and diabetes. Notably $H_{2}$ may be produced in gut by bacteria in the human body. This process can be optimized through dietary choices, particularly the MD which is rich in fiber and bioactive compounds. Given the growing body of evidence supporting $H_{2}$’s positive impact on metabolic and cardiovascular conditions, targeted strategies to increase intestinal $H_{2}$ production may serve as future preventive measures or adjunctive treatments of these diseases.
